# Identification of Selection Signatures and Candidate Genes Related to Environmental Adaptation and Economic Traits in Tibetan Pigs

**DOI:** 10.3390/ani14040654

**Published:** 2024-02-19

**Authors:** Pengliang Liu, Yan Liang, Li Li, Xuebin Lv, Zhiping He, Yiren Gu

**Affiliations:** 1 Key Laboratory of Qinghai-Tibetan Plateau Animal Genetic Resource Reservation and Utilization, Ministry of Education, Southwest Minzu University, Chengdu 610041, China; pengliangliu@swun.edu.cn; 2 Animal Breeding and Genetics Key Laboratory of Sichuan Province, Sichuan Animal Science Academy, Chengdu 610066, China; lwenwen11@163.com (Y.L.);; 3 Renshou County Bureau of Agriculture and Rural Affairs, Meishan 620500, China

**Keywords:** Tibetan pig, environmental adaptation, economic traits, SNP, candidate genes

## Abstract

**Simple Summary:**

Modern agricultural methods have led to the introduction of normally highland Tibetan pigs to lowland areas because of their high-quality meat. Similar to humans, indigenous Tibetan pigs residing in high-altitude regions may develop high-altitude de-acclimatization syndrome when exposed to low-altitude environments. Thus, it is important to better understand the low-altitude adaptation mechanisms of Tibetan pigs. We performed a comparative analysis between highland and lowland Tibetan pigs using genotyping data to investigate their genetic differences. Our results showed that immune-related genes were selected in highland Tibetan pigs, whereas genes associated with reproduction, growth and development, and blood pressure regulation were selected in lowland Tibetan pigs. These findings will contribute to research into the genetic basis of environmental adaptation and important economic traits in Tibetan pigs and provide genetic targets for breeding programs to improve Tibetan pigs.

**Abstract:**

Tibetan pigs are indigenous to the Qinghai–Tibet Plateau and have been the subject of extensive genomic research primarily focused on their adaptation to high altitudes. However, genetic modifications associated with their response to low-altitude acclimation have not been thoroughly explored. To investigate the genetic basis underlying the low-altitude acclimation of Tibetan pigs, we generated and analyzed genotyping data of Tibetan pigs that inhabit high-altitude regions (average altitude 4000 m) and Tibetan pigs that have inhabited nearby low-altitude regions (average altitude 500 m) for approximately 20 generations. We found that the highland and lowland Tibetan pigs have distinguishable genotype and phenotype variations. We identified 46 and 126 potentially selected SNPs associated with 29 and 56 candidate genes in highland and lowland Tibetan pigs, respectively. Candidate genes in the highland Tibetan pigs were involved in immune response (*NFYC* and *STAT1*) and radiation (*NABP1*), whereas candidate genes in the lowland Tibetan pigs were related to reproduction (*ESR2*, *DMRTA1*, and *ZNF366*), growth and development (*NTRK3*, *FGF18*, and *MAP1B*), and blood pressure regulation (*CARTPT*). These findings will help to understand the mechanisms of environmental adaptation in Tibetan pigs and offer valuable information into the genetic improvement of Tibetan pigs pertaining to low-altitude acclimation and economic traits.

## 1. Introduction

Tibetan pigs are a typical plateau-type pig breed in China. They are distributed primarily in the Qinghai–Tibet Plateau region, which has an average altitude of 4000 m. Millions of years of natural selection have allowed Tibetan pigs to evolve unique biological characteristics to survive in the harsh plateau environment, including strong adaptability to low-oxygen levels, low temperatures, low pressure, strong ultraviolet radiation, and high resistance to diseases. Tibetan pigs have better meat quality and flavor than Western pigs and are superior to other Chinese indigenous pigs in some respects. For instance, Gan et al. found that Tibetan pigs had a higher proportion of essential fatty acids and umami amino acids than Yorkshire pigs and Qingyu pigs (a typical indigenous Chinese pig breed) [1]. Although Tibetan pigs are known for their excellent meat quality, they lack specialized breeding and continuous improvement. Their growth and reproduction rates are lower than those of Western pig breeds and those of commercial pigs. Therefore, improving the growth rate and fecundity of Tibetan pigs is a key point for future Tibetan pig breeding programs.

Natural and artificial selection have left detectable footprints in the genome of Tibetan pigs. A comparative genome analysis between wild Tibetan pigs and Duroc pigs found that many genes associated with hypoxia response, DNA repair, and energy metabolism had undergone strong natural selection in Tibetan pigs [2]. The genetic mechanism of high-altitude adaptation of Tibetan pigs was discovered using selection signature detection methods based on single-nucleotide polymorphisms (SNPs). For instance, Ai et al. identified several candidate genes, *PLA2G12A*, *RGCC*, *C9ORF3*, *GRIN2B*, *GRID1*, and *EPAS1*, that contributed to high-altitude adaptation in Tibetan pigs [3]. Ma et al. identified 273 potentially positively selected genes in the Tibetan pig genome by comparing their genomes with the genome of lowland domestic pigs in China, including genes related to hypoxia and circulation (e.g., *CYP4F2*, and *THSD7A*) [4]. Dong et al. discovered 12 specific selective genes (*CCBE1*, *F2RL1*, *AGGF1*, *ZFPM2*, *IL2*, *FGF5*, *PLA2G4A*, *ADAMTS9*, *NRBF2*, *JMJD1C*, *VEGFC*, and *ADAM19*) in Tibetan pigs by comparing them with Wuzhishan pigs (low-altitude breed) [5]. Together, these two previous studies show that genomic comparisons can contribute to the genetic resolution of important traits in Tibetan pigs [4,5]. However, most genetic studies of Tibetan pigs have concentrated primarily on traits associated with high-altitude adaptation, and other traits have been poorly studied.

Modern agriculture methods have led to the introduction of Tibetan pigs into lowland areas because of their high-quality meat. However, normal air pressure and sufficient oxygen can lead to hyperoxia, causing oxygen toxicity or oxygen poisoning in Tibetan pigs. Therefore, Tibetan pigs from highland areas underwent long periods of domestication to adapt effectively to lowland environments. Nevertheless, the genetic basis of Tibetan pig adaptation to lowland environments remains largely unknown. We obtained a Tibetan pig population that had undergone nearly 20 generations of lowland acclimation (average altitude 500 m) and artificial selection for growth and reproduction. This population provides a unique opportunity to explore the genetic basis of low-altitude acclimation and improvements in the economic traits of Tibetan pigs. In this study, we explored the population structure between highland (average altitude 4000 m) and lowland (average altitude 500 m) Tibetan pigs and identified genomic regions that had undergone selection in each population using porcine SNP chips. Our results will help further understand the genetic mechanisms of the high- and low-altitude environmental adaptation of Tibetan pigs and provide reference data for the genetic improvement of Tibetan pigs in the future.

## 2. Materials and Methods

### 2.1. Ethics Approval

All the animal studies were conducted according to the guidelines for the Care and Use of Experimental Animals established by the Ministry of Agriculture and Rural Affairs of China.

### 2.2. Animals and Sample Preparation

A total of 149 healthy 2-year-old female Ganzi Tibetan pigs were used in this study. They included 100 Tibetan pigs from the Tibetan highland region (Daocheng, Sichuan, China), which has an average altitude of 4000 m, and 49 Tibetan pigs from the geographically neighboring lowland region (Chengdu, Sichuan, China), which has an average altitude of 500 m. The lowland Tibetan pigs are descendants of highland Ganzi Tibetan pigs (Daocheng, Sichuan, China) that have inhabited the low-altitude region for nearly 20 generations. All these pigs were single-housed and were allowed ad libitum access to feed and water under the same feeding conditions. Moreover, we also collected samples from healthy 1-year-old female Chenghua pigs and Neijiang pigs, which were used as the outgroup. Ear tissue (approximately 0.5 g) was collected from each pig, preserved in 75% ethanol, and stored at −20 °C until DNA extraction.

### 2.3. Determination of Phenotypic Characteristics

Two important economic traits, backfat thickness and average daily weight gain, for 149 Tibetan pigs (100 highland Tibetan pigs and 49 lowland Tibetan pigs) were measured in this study. Specifically, the backfat thickness of each living Tibetan pig was measured in accordance with the Chinese Guidelines on Performance Measurement Technology and Regulations for Pigs, when the Tibetan pig grew in body weight up to 50 kg. It was measured on the left side, specifically between the last third and fourth ribs, at a distance of 5 cm from the dorsal line, utilizing a real-time B-mode ultrasound machine. The average daily weight gain was measured during the period in which the pig grew from a body weight of 20 to 50 kg. In brief, the ages of pigs were recorded when the weight reached 20 and 50 kg, and the average weight daily gain was calculated by dividing the weight gain by the number of days from 20 to 50 kg. Thus, the average daily weight gain was calculated as

$$Average\;daily\;weight\;gain\;(kg/d)=\frac{50-20}{age\;50\;kg\;(day)-age\;20\;kg\;(day)}$$

### 2.4. DNA Extraction

Genomic DNA was extracted from the ear tissue using a DNA extraction kit (TIANGEN, Beijing, China, DP304) according to the manufacturer’s instructions. The extracted DNA was quantified and quality-checked using a Nanodrop 2000 spectrophotometer. Only DNA samples with concentrations > 50 ng µL^−1^ and 1.7 < OD_260nm_/OD_280nm_ < 2.0 were used for further analysis.

### 2.5. Genotyping and Quality Control

Genotyping was performed using Zhongxin-1 Porcine Breeding Array PLUS chips (Compass Biotechnology Co., Ltd., Beijing, China), which contain 57,466 SNP markers. Quality control for the raw genotype data was conducted using PLINK (v1.9) software [6]. SNPs with call rates < 90% were excluded, and SNPs that were located in non-autosomal chromosomes or had a minimum allele frequency < 0.05 or Hardy–Weinberg equilibrium test *p*-value < 1.0 × 10^−6^ were filtered out. After filtering, 18,254 autosomal SNPs were retained for subsequent analyses.

### 2.6. Population Structure and Phylogenetic Analysis

To investigate the population structure in our data set, we performed a principal component analysis (PCA) of the 18,254 high-quality SNPs using PLINK. The ggplot package in R was used to visualize the clustering of samples with the first two principal components (PC1 and PC2). To further explore the relationships between individuals, a pairwise identity-by-state (IBS) distance matrix was calculated between individuals using PLINK. Then, a population neighbor-joining tree was constructed based on the IBS distance matrix using MEGA (v10) software [7]. Finally, the population genetic structure was constructed using Admixture (v1.3) software [8] (K = 2) to examine the Admixture level among individuals. The Admixture results were visualized using the pophelper package in R.

### 2.7. Linkage Disequilibrium Analysis

Linkage disequilibrium (LD) values for each population were calculated for 100 kb windows based on the squared correlation coefficient (*r*^2^) between each pair of SNPs using PopLDdecay (v3.4) software [9] with default parameters. The decay of LD with physical distance between SNPs was visualized using PopLDdecay [8].

### 2.8. Detection of Selection Signatures and Gene Annotation

To investigate the genetic differentiation between the highland and lowland Tibetan pigs, the population differentiation fixation index (*F*_ST_) and nucleotide polymorphism levels (*θ*_π_) were calculated using VCFtools [10] (v0.1.17) (5 Mb window sliding in 100 kb steps). *F*_ST_ values were calculated as

*F*_ST_ = (Ht − Hs)/Ht,

where Hs is the expected average heterozygosity in subpopulations and Ht is the expected heterozygosity of the entire population.

To identify the genomic regions with significant signatures of selective sweep, we examined the distribution of *F*_ST_ values and *θ*_π_ ratios (*θ*_π,highland_/*θ*_π,lowland_). Min–max normalization was applied to visualize the distribution of *F*_ST_ values and *θ*_π_ ratios. The original (unscaled) *F*_ST_ values and *θ*_π_ ratios were scaled to a range of [0, 1], as follows:

$$y_{i}=\frac{x_{i}-\underset{1\leq j\leq n}{\min}\left\{x_{j}\right\}}{\underset{1\leq j\leq n}{\max}\left\{x_{j}\right\}-\underset{1\leq j\leq n}{\min}\left\{x_{j}\right\}}$$

Take *F*_ST_ values as an example. $y_{i}$ represents the scaled *F*_ST_ value for the *i*th genomic region in the genome. $x_{i}$ and $x_{j}$ represent original (unscaled) *F*_ST_ values for the *i*th and *j*th genomic regions in the genome, respectively. Thus, $\underset{1\leq j\leq n}{\max}\left\{x_{j}\right\}$ and $\underset{1\leq j\leq n}{\min}\left\{x_{j}\right\}$ represent the maximum and minimum values of the *F*_ST_ values across the genome, respectively. The normalization of the *θ*_π_ ratios was performed following the same procedure.

We selected the windows with significant low and high *θ*_π_ ratios (the 1% top and 1% bottom, where the *θ*_π_ ratios [*θ*_π,highland_/*θ*_π,lowland_] were 1.308 and 0.804, respectively) and significant high *F*_ST_ values (the 1% top, where the *F*_ST_ value was 0.170) as the genomic regions with selective sweep signals along the genome. The SNPs in these regions were defined as candidate SNPs and were annotated using the ANNOVAR tool. The annotated genes were defined as candidate genes.

### 2.9. Functional Enrichment Analysis

To better understand the functions and signaling pathways of the candidate genes, we performed Gene Ontology (GO) and Kyoto Encyclopedia of Gene and Genomes (KEGG) enrichment analyses based on a hypergeometric test. GO terms and KEGG pathways with *p*-values < 0.05 were considered to be significantly enriched.

## 3. Results and Discussion

### 3.1. Detection of SNPs between Highland and Lowland Tibetan Pigs

**Figure 1:**
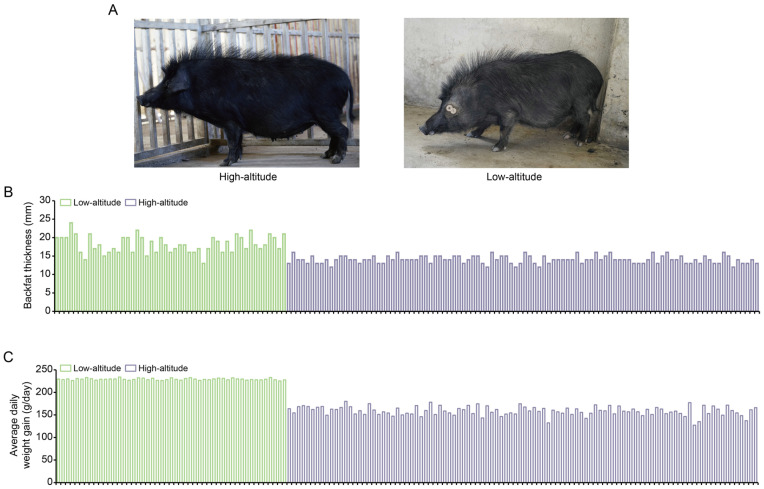
Phenotypic comparison between high-altitude and low-altitude Tibetan pigs. (**A**) Appearance of the 1-year-old female high-altitude (**left**) and low-altitude (**right**) Tibetan pigs. (**B**,**C**) Comparison of backfat thickness (**B**) and average daily weight gain (**C**) between high-altitude (*n* = 100) and low-altitude (*n* = 49) Tibetan pigs.

**Figure 2:**
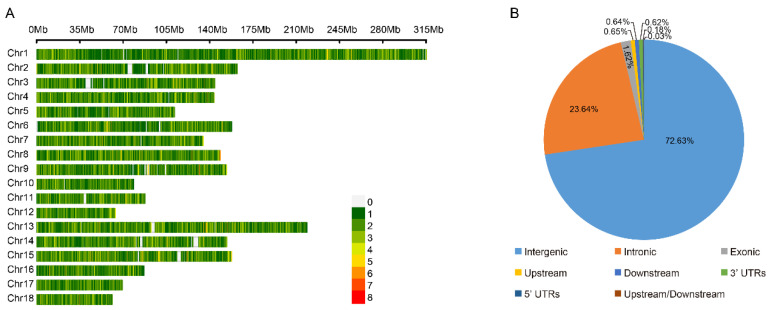
The distribution of SNPs detected in high-altitude and low-altitude Tibetan pigs. (**A**) Genome-wide distribution of detected SNPs on 18 autosomes chromosomes in the pig genome. Calculated as the number of SNPs per 0.1 Mb. (**B**) Distribution of detected SNPs in different genomic elements in the pig genome. ‘Upstream’ regions were defined as the 1 kb region upstream from the gene start site. ‘Downstream’ regions were defined as the 1 kb region of the gene end site. ‘Upstream/Downstream’ indicate a variant located in the downstream and upstream regions (might be two different genes).

We compared the phenotypical features of the lowland Tibetan pigs that have inhabited a low-altitude region (500 m) for nearly 20 generations with those of the highland Tibetan pigs that inhabit a high-altitude region (4000 m). In terms of appearance, we found that the lowland Tibetan pigs have less hair on their bodies when compared with the highland Tibetan pigs (Figure 1A). Due to the poor ability of the pig to dissipate heat, thick hair can intensify the adverse impacts of high temperatures in low-altitude regions, potentially resulting in heat stress. Therefore, a reduction in hair on their body surface may represent an adaptive change of lowland Tibetan pigs to their lowland habitat. Furthermore, compared with the highland Tibetan pigs, the lowland Tibetan pigs had higher back fat thickness (median 14 vs. 18 mm) and higher average daily weight gain (median 159 vs. 229 g/pig per day) with *p*-values < 2.2 × 10^−16^ according to unpaired two-tailed Student’s *t*-tests (Figure 1B,C). This finding confirms that artificial selection is an important method for improving the productive performance of Tibetan pigs. To investigate genotypic differences between the 100 highland and 49 lowland Tibetan pigs, they were successfully genotyped using Zhongxin-1 Porcine Breeding Array PLUS chips. A total of 45,212 SNPs were identified, and after applying the QC filters (see the Materials and Methods section for details), we obtained 18,254 autosomal SNPs for further analysis. These SNPs were evenly distributed across their chromosomes (Figure 2A), and most of them were located in intronic (*n* = 4316, 23.64%) or intergenic (*n* = 13,258, 72.63%) regions (Figure 2B). The number of identified SNPs was less than the numbers identified in previous comparisons between the highland Tibetan pigs and the lowland Chinese indigenous pig breeds [3,5]. Two main explanations for this discrepancy are possible. First, we used chips for the SNP genotyping, which may have missed SNPs that were detected by whole-genome sequencing. Nevertheless, previous studies have shown that the Zhongxin-1 Porcine Breeding Array PLUS chip provided reliable estimates of genomic diversity and selection signatures in a comparative analysis between pig breeds [11]. Second, low-altitude Chinese indigenous pig breeds have considerable phenotype diversity due to the thousands of years of domestication and are therefore more genetically differentiated than the high-altitude Tibetan pigs. Importantly, the low-altitude Tibetan pigs used in this study have only experienced domestication and artificial selection in lowland regions for approximately 20 generations, and therefore, there is relatively little genetic difference between them and the high-altitude Tibetan pigs.

### 3.2. Population Structure Analysis

**Figure 3:**
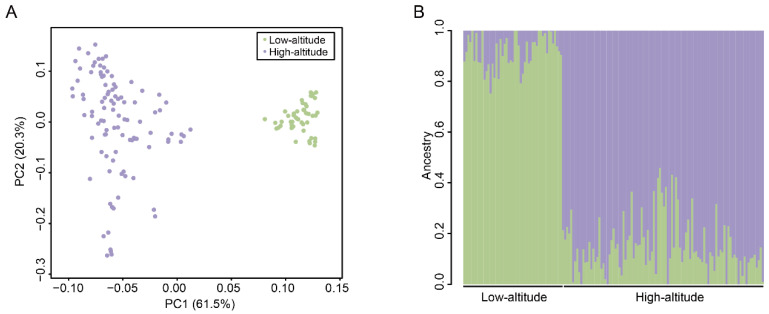
Population structure of 149 Tibetan pigs. (**A**) The PCA plots of the high-altitude (purple) and low-altitude (green) samples based on the genotyped SNPs. (**B**) Population structure inferred by Admixture analysis based on identified SNPs. The different colors indicate different ancestral populations. Each small vertical line represents an individual.

We performed a PCA to determine the clustering patterns of SNP genotypes among the pigs. The two pig populations were distinctly separated by the first principal component (PC1), which explained 61.5% of the overall variance (Figure 3A). This finding confirmed that there were differences between the highland and lowland Tibetan pigs at the genomic sequence level. Furthermore, individuals in the lowland Tibetan pig population are more aggregated in the PCA plot than individuals in the highland population (Figure 3A), which may be related to the strong artificial selection that occurs in the lowland Tibetan pig population. Next, we conducted an Admixture analysis, and the results remarkably distinguished the two pig populations, which is consistent with the PCA results (Figure 3B). Together, these results confirm that the genotypes of highland and lowland Tibetan pigs are different. To better reflect the relationship between the highland and lowland Tibetan pigs, we supplemented the genotype data of two Chinese indigenous breed (Chenghua pig and Neijiang pig). The PCA result showed that the Tibetan pigs, Chenghua pigs, and Neijiang pigs were separated by PC1, whereas the highland and lowland Tibetan pigs were separated by PC2 (Supplementary Figure S1). A neighbor-joining (NJ) tree showed that the all individuals are clustered under their respective populations (Supplementary Figure S2). The highland Tibetan pig population is close to the lowland Tibetan pigs but far from the Chenghua and Neijiang pigs (Supplementary Figure S2), indicating the close relationship between the highland and lowland Tibetan pigs. Thus, even though there are some differences between the highland and lowland Tibetan pigs at the genotype level, these differences are smaller than those among Tibetan pigs and the other two Chinese indigenous breeds.

### 3.3. Linkage Disequilibrium Analysis

**Figure 4:**
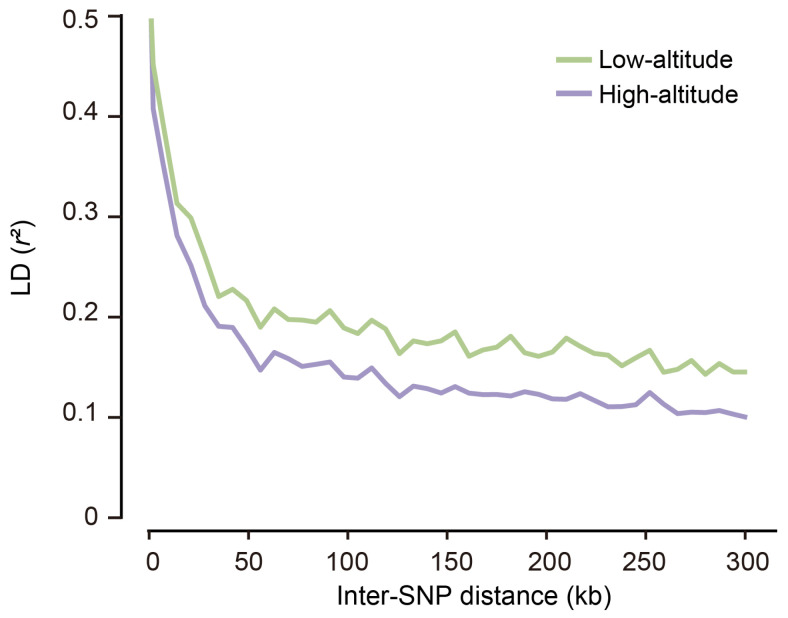
Extent of LD (*r*^2^) as a function of physical distance between SNPs within each population. The samples of both populations used for the LD analysis was equal (*n* = 49).

To estimate the extent of LD for the highland and lowland Tibetan pig populations, we calculated the squared correlation coefficient (*r*^2^) between pairs of SNPs. We found that the LD extent decreased rapidly with increasing physical distance between SNPs and that similar patterns exist in both populations (Figure 4), which is consistent with the results of other studies [12,13,14]. However, the rate of LD decay varied between populations; the lowland Tibetan pigs had stronger LD than the highland Tibetan pigs. This finding suggests that the lowland Tibetan pigs had experienced stronger selection than the indigenous highland Tibetan pig. To evaluate the patterns of LD extent, *r*^2^_0.3_ values were calculated to represent the physical distance at which the genotypic association between pairs of SNPs was below a threshold of 0.3. The *r*^2^_0.3_ values for the highland and lowland Tibetan pigs were <20 kb, which is lower than the distances previously found in Western domestic pigs (e.g., Duroc, 413 kb; Landrace, 334 kb; Large White, 280 kb; and White Duroc, 757 kb) and Chinese domestic pigs (e.g., Bamaxiang, 143 kb; Dongshan, 311 kb), and similar to that in Chinese wild boar (38 kb) [15]. It is well established that selection elevates the LD extent for a population [16,17]. Our findings suggest that Tibetan pigs were potentially subjected to less selective pressure and therefore retain more genetic diversity than Western commercial pigs and most other Chinese local pigs.

### 3.4. Selection Signature Detection and Identification of Candidate Genes

**Figure 5:**
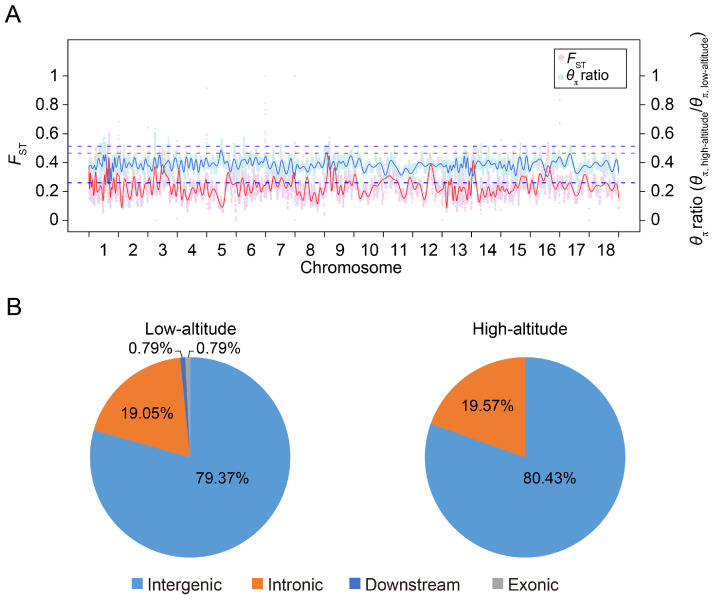
Distribution of putatively selective signatures in the high-altitude and low-altitude Tibetan pigs on the 18 autosome chromosomes. (**A**) The *F*_ST_ values and *θ*_π_ ratios (*θ*_π,highland_/*θ*_π,lowland_) across the pig genome. The *F*_ST_ values and *θ*_π_ ratios were scaled by min–max normalization. The pink and light blue points represent the *F*_ST_ values and *θ*_π_ ratio for the genomic regions, respectively. The red and blue lines are the trend lines for the *F*_ST_ values and *θ*_π_ ratio, respectively. The red dashed line represents the top 1% rank value of *F*_ST_ values, while the blue dashed lines represent the top and bottom 1% values of *θ*_π_ ratios (*θ*_π,highland_/*θ*_π,lowland_), respectively. (**B**) Distribution of candidate SNPs in high-altitude and low-altitude Tibetan pigs in different genomic elements in the pig genome. ‘Downstream’ regions were defined as the 1 kb region of the gene end site.

To investigate selective signatures between the highland and lowland Tibetan pigs, we examined the distribution of *F*_ST_ values and relative diversity of nucleotide polymorphism levels (*θ*_π_ ratio, highland/lowland) between the two populations. We identified thirty-eight 5 Mb windows with strong selective sweep signals (defined as putative selective regions) in highland Tibetan pigs and eighty-three 5 Mb windows in lowland Tibetan pigs (see the Materials and Methods section for details). A total of 46 and 126 SNPs were located in the putative selective regions of highland and lowland Tibetan pigs, respectively, and were considered as candidate SNP loci (Table S1). This result again supports the stronger selective pressure in the lowland Tibetan pig population compared with the selective pressure in the highland Tibetan pig population. The genomic distribution of candidate SNP loci is shown in Figure 5. It is worth noting that almost all candidate SNPs were located within intronic and intergenic regions, particularly within the intergenic regions. The intergenic candidate SNPs account for about 80% of all candidate SNPs. In fact, many *cis* regulatory elements (such as enhancers) are mainly localized to the intergenic regions in the pig genome [18]. Recent investigations have additionally demonstrated a noteworthy enrichment of GWAS significantly associated SNPs and selection signatures within cis regulatory elements, particularly promoters and enhancers, in the pig genome [19,20]. Moreover, SNPs possess the potential to impact gene expression by disrupting the functionality of *cis* regulatory elements [21]. Consequently, we hypothesized that the regulation of our identified candidate SNPs is primarily mediated by the *cis* regulatory elements located within intergenic regions.

**Figure 6:**
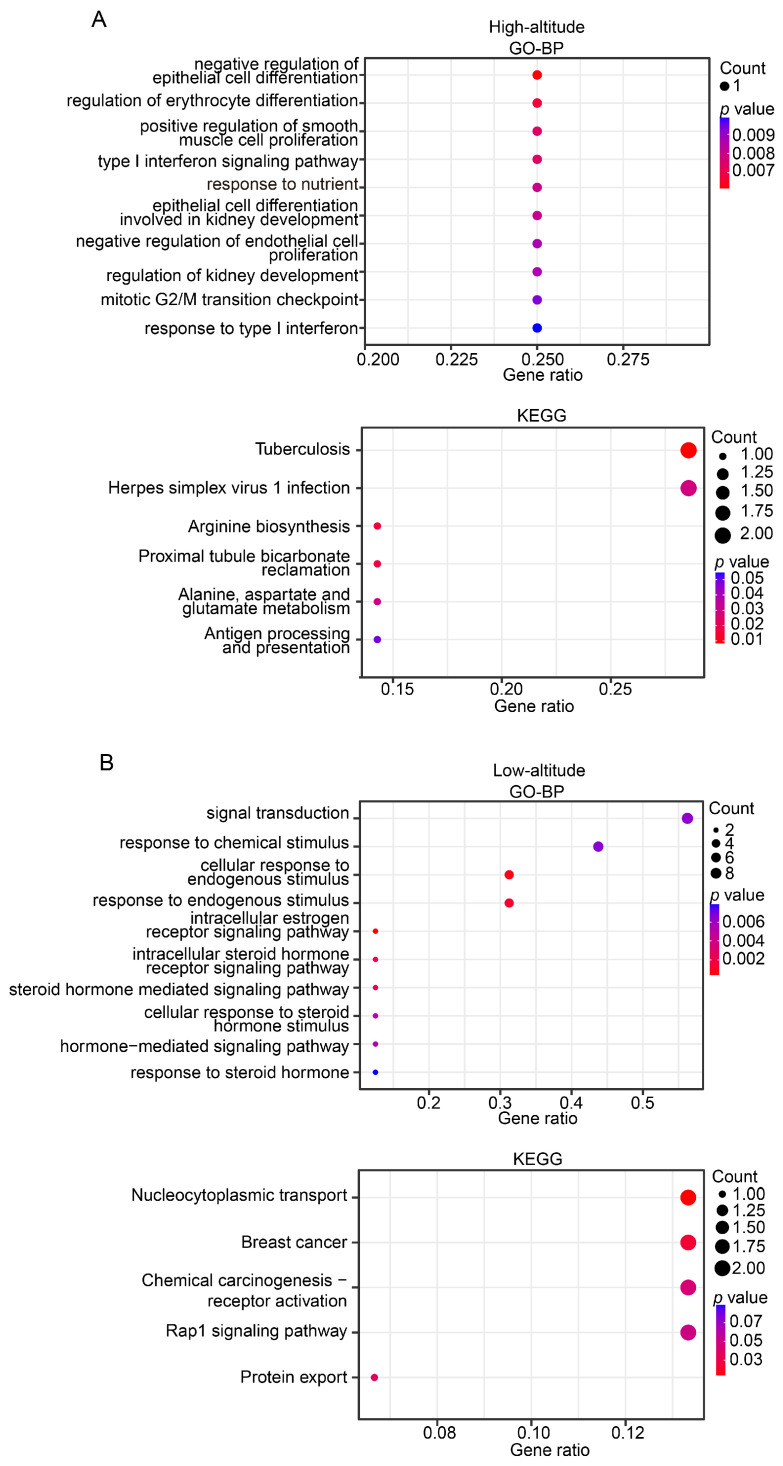
Functional enrichment for identified candidate genes in high-altitude (**A**) and low-altitude (**B**) Tibetan pigs. Plot showing the most statistically significant (up to 10) GO terms (upper panel) and KEGG pathways (bottom panel). Dot size indicates the gene number, and the dot color represents the *p* value. The x-axis represents the proportion of identified candidate genes for a given GO term or KEGG pathway.

The gene annotation helped identify 29 and 56 candidate genes in the highland and lowland populations, respectively (Table S2). The GO functional enrichment analysis showed that the candidate genes in the highland population were associated with a broad spectrum of biological functions, including erythrocyte differentiation, immune, development, and metabolism (Figure 6A). Remarkably, the KEGG analysis showed that three of six significantly enriched pathways (hypergeometric test, *p*-value < 0.05) were immune-related, namely tuberculosis, herpes simplex virus 1 infection, and antigen processing and presentation (Figure 6A). These findings are consistent with the general observation that Tibetan pigs may be more resistant to disease and have a low incidence of disease during growth [22].

We manually checked the biological functions of the candidate genes identified in the highland Tibetan pig population. A functional enrichment analysis indicated that two of the genes (*NFYC* and *STAT1*) encode transcription factors that are closely associated with immune function. Transcription factors have widespread roles in gene regulation, which suggests that these two genes may be important in modulating immune responses. The STAT1 protein plays a crucial roles in both innate and acquired immune responses by transducing the signal from interferons and other cytokines (e.g., interleukins) in the nucleus [23,24,25], thereby protecting the host against viruses and other pathogens [26,27,28,29]. The deficiency of STAT1 in mice made them highly susceptible to bacterial and viral infections, ultimately leading to death [30,31,32]. The NFYC protein binds to the promoter of major histocompatibility complex (MHC) genes and modulates their transcription [33,34], and therefore plays important roles in immune responses against viruses and pathogens. Besides these two genes, several candidate genes related to high-altitude adaptation were identified (e.g., *NABP1*, which may be involved in the adaptation to radiation from high altitudes). Interestingly, candidate genes related to hypoxia adaptation were rarely found, implying that the lowland Tibetan pigs may still retain the hypoxia adaptation.

We also examined the candidate genes identified in the lowland Tibetan pig population. A functional enrichment analysis indicated that these genes were enriched mainly in GO enrichment categories related to estrogen signaling (e.g., intracellular estrogen receptor signaling pathway and intracellular steroid hormone receptor signaling pathway) (Figure 6B), suggesting that the candidate genes were likely to be closely associated with reproduction. We identified three genes (*ESR2*, *DMRTA1*, and *ZNF366*) that may be related to reproduction. ESR2 plays a critical role in folliculogenesis and ovulation, and the disruption of its function in rats resulted in female infertility due to failure of ovulation [35,36]. *Dmrta1* may also play important roles in primordial follicle formation because the targeted deletion of *Dmrta1* caused multi-oocyte follicles [37]. Three other genes (*NTRK3* [38,39], *FGF18* [40,41], and *MAP1B* [42]) that are related to growth and development were identified, which is consistent with the observed higher back fat thickness and daily weight gain in the lowland Tibetan pigs compared with the highland Tibetan pigs.

Indeed, although Tibetan pigs are known for their good meat quality, they have a slower growth rate and lower fecundity than Western pig breeds and most indigenous Chinese pig breeds. Therefore, it is imperative to improve the growth rate and reproduction performance of Tibetan pigs to further expand their economic value. The growth- or reproduction-related candidate genes and SNPs identified in this study are potential molecular genetic targets for Tibetan pig breeding programs. We also identified a gene (*CARTPT*) involved in the regulation of blood pressure and blood circulation. A previous study has demonstrated that the chemogenetic activation of the CART (encode by *Cartpt* gene) neuron population can lead to reductions in blood pressure and heart rate in mice [43]. Indeed, previous studies have indicated that alterations in altitude can impact the blood pressure and heart rate of mammalian organism. For instance, one study observed changes in the resting systolic blood pressure, diastolic blood pressure, and heart rate of low-altitude migrants during a 1-year stay at high altitude. Their systolic blood pressure and diastolic blood pressure increased following short-term exposure to high altitudes but were lower than the control values in prolonged exposure (180–360 day) [44]. Likewise, when the high-altitude Tibetan natives were exposed to normobaric normoxic environments, their heart rate and blood pressure also changed. Therefore, we considered that the *CARTPT* gene may be implicated in the low-altitude adaptation of Tibetan pigs. Altogether, the functions of these discussed candidate genes deserve further research attention, and the haplotypes around these genes can be found in Table S3.

## 4. Conclusions

We conducted a comparative analysis of genotyping data between highland and lowland Tibetan pigs. We showed that candidate genes related to immune response (*NFYC* and *STAT1*) and radiation (*NABP1*) were potentially selected in highland Tibetan pigs, which is consistent with their superiority in highland adaptation and resistance to disease. Candidate genes associated with growth and development (*NTRK3*, *FGF18*, and *MAP1B*) and reproduction (*ESR2*, *DMRTA1*, *ZNF366*) were potentially selected in lowland Tibetan pigs, which is consistent with their outstanding performance in growth and reproduction. A candidate gene involved in blood pressure regulation (*CARTPT*) was also selected in lowland Tibetan pigs, which may be related to the low-altitude adaptation in lowland Tibetan pigs.

## Data Availability

The genotype data sets generated in this study are available in FigShare (https://doi.org/10.6084/m9.figshare.24721341.v1, accessed on 15 February 2024; https://doi.org/10.6084/m9.figshare.25222799.v1, accessed on 15 February 2024).

## Supplementary Materials

The following supporting information can be downloaded at https://www.mdpi.com/article/10.3390/ani14040654/s1, Table S1. Candidate SNPs identified in high-altitude and low-altitude Tibetan pigs. Table S2. Candidate genes identified in high-altitude and low-altitude Tibetan pigs. Table S3. The haplotypes around the representative genes based on their associated candidate SNPs. Figure S1. The PCA plots of Chenghua pigs, Neijiang pigs, high-altitude Tibetan pigs, and low-altitude Tibetan pigs based on genotyped SNPs. Figure S2. Neighbor-joining tree of the four pig populations.
