# Supplementary material for: Identification of Selection Signatures and Candidate Genes Related to Environmental Adaptation and Economic Traits in Tibetan Pigs
Source: Animals (Basel). 2024 Feb 19;14(4):654. doi: 10.3390/ani14040654 (PMC10886212; doi:10.3390/ani14040654)
Supplement: Supplementary file 1 [file animals-14-00654-s001.zip › Supplementary figures.pdf]

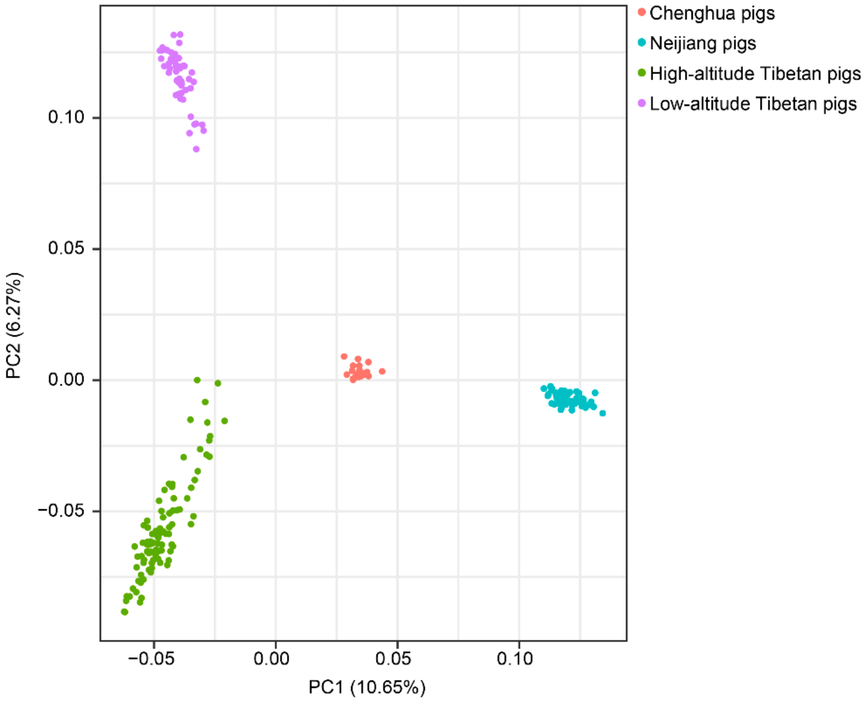

Figure S1. The PCA plots of Chenghua pigs, Neijiang pigs, high-altitude Tibetan pigs and low-altitude Tibetan pigs based on genotyped SNPs.

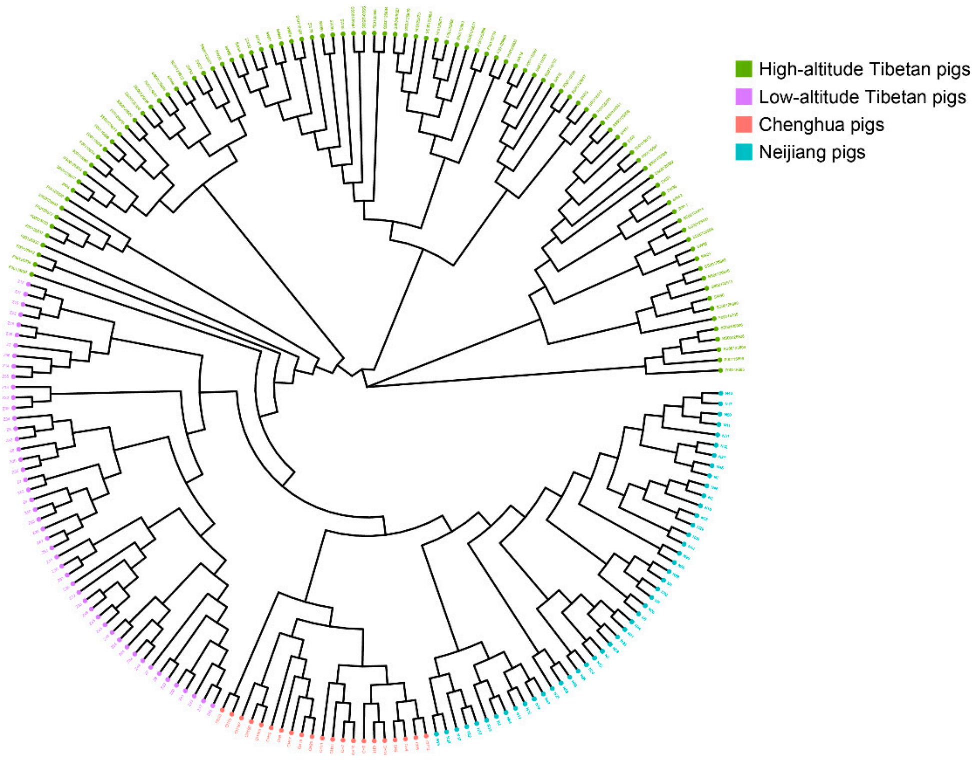

Figure S2. Neighbor-joining tree of the four pig populations.
